# Photothermal spectroscopy on-chip sensor for the measurement of a PMMA film using a silicon nitride micro-ring resonator and an external cavity quantum cascade laser

**DOI:** 10.1515/nanoph-2024-0033

**Published:** 2024-03-15

**Authors:** Giovanna Ricchiuti, Anton Walsh, Jesús Hernán Mendoza-Castro, Artem S. Vorobev, Maria Kotlyar, Gustavo V. B. Lukasievicz, Simone Iadanza, Marco Grande, Bernhard Lendl, Liam O’Faolain

**Affiliations:** 27259 Institute of Chemical Technologies and Analytics, TU Wien, Getreidemarkt 9/164, 1060, Vienna, Austria; Centre for Advanced Photonics and Process Analysis, 587895 Munster Technological University, T12 T66T Bishopstown, Cork, Ireland; Tyndall National Institute, T12 PX46 Cork, Ireland; Department of Electrical and Information Engineering, Polytechnic University of Bari, 4, 70126 Bari, Italy; Department of Physics, Federal University of Technology-Paraná, Medianeira PR 85884-000, Brazil; Laboratory of Nano and Quantum Technologies Paul-Scherrer-Institut ODRA/114 Forschungsstrasse 111 – 5232 Villigen PSI Schweiz, Villigen, Switzerland; Laboratory of Integrated Nanoscale Photonics and Optoelectronics, Ecole Polytechnique Federale de Lausanne, Lausanne, Switzerland

**Keywords:** photothermal spectroscopy, mid-infrared spectroscopy, photonics integrated circuits, micro-ring resonator, sensing, on-chip sensor

## Abstract

Laser-based mid-infrared (mid-IR) photothermal spectroscopy (PTS) represents a selective, fast, and sensitive analytical technique. Recent developments in laser design permits the coverage of wider spectral regions in combination with higher power, enabling for qualitative reconstruction of broadband absorption features, typical of liquid or solid samples. In this work, we use an external cavity quantum cascade laser (EC-QCL) that emits in pulsed mode in the region between 5.7 and 6.4 µm (1770–1560 cm^−1^), to measure the absorption spectrum of a thin film of polymethyl methacrylate (PMMA) spin-coated on top of a silicon nitride (Si_3_N_4_) micro-ring resonator (MRR). Being the PTS signal inversely proportional to the volume of interaction, in the classical probe–pump dual beam detection scheme, we exploit a Si_3_N_4_ transducer coated with PMMA, as a proof-of-principle for an on-chip photothermal sensor. By tuning the probe laser at the inflection point of one resonance, aiming for highest sensitivity, we align the mid-IR beam on top of the ring’s area, in a transversal configuration. To maximize the amplitude of the photoinduced thermal change, we focus the mid-IR light on top of the ring using a Cassegrain reflector enabling for an optimal match between ring size and beam waist of the excitation source. We briefly describe the transducer design and fabrication process, present the experimental setup, and perform an analysis for optimal operational parameters. We comment on the obtained results showing that PTS allows for miniaturized robust sensors opening the path for on-line/in-line monitoring in several industrial processes.

## 1 Introduction

Waveguide (WG)-based devices are increasingly gaining favor within the field of optical signal processing for sensing purposes across various domains, notably in the scope of chemical and bio-chemical detection [1], [2]. Photonic sensors have the capability to identify fluctuations in the refractive index (RI), absorption, or fluorescence of a substance under scrutiny, enabling the correlation of these changes with the concentration of a target molecule or the presence of a specific substance [3]. Among the advantages of WG sensors, there are high compactness, low-costs in the fabrication process, and energy efficiency [4].

Optical RI sensors are based on a variation of the effective refractive index (*n*_eff_) of the propagating optical mode due to a certain substance placed at the sensor outer interface. The design of integrated photonic sensors is thus optimized to enhance the sensitivity of such devices by tuning the geometry/shape, the operational wavelength, and choosing the proper materials based on the eventual application and compatibility.

In particular, micro-ring resonator-based sensors, fabricated from complementary metal-oxide-semiconductor (CMOS) compatible materials, such as silicon (Si) and silicon nitride (Si_3_N_4_), have gained growing interest due to their high sensitivity, small footprint, and low fabrication costs [5], [6], [7], [8], [9]. In literature, MRRs, used as temperature and/or refractive index sensors [5], [6], [7], [8], [9], permit to measure a specific molecule at different concentrations by estimating the resonant wavelength shift (Δ*λ*_Res_) due to the introduced refractive index change (Δ*n*). In fact, the ratio between Δ*λ*_Res_ and Δ*n* gives an estimation of the waveguide sensitivity *S*_*w*_ of the device expressed in [nm/Refractive Index Unit (RIU)]. Unfortunately, if on the one hand, those micro-transducers are promising for sensing purposes, then on the other hand, they exhibit only limited selectivity meaning that a shift of the *λ*_Res_ will occur independently on the target analyte and/or the interfering molecules. The sensor response is not capable of discrimination. To overcome this limitation, one could resort to surface functionalization [10], [11], where binding agents are deposited on the surface, based on the ultimate application, to regulate certain functions both spatially and temporarily [12], [13], [14], [15]. Clearly, this additional process requires some time before operating the sensor that can be used later only for the specific application for which it was prior functionalized. Additionally, the process could affect the physical and chemical response of the surface and the result could fade off after limited amount of time.

In laser spectroscopy, tunable laser sources are employed for selective excitation of molecular species. However, classical absorption approach would not benefit from smaller sample size limited by the area of the WG-based sensor itself (few µm in general) with which the sample is in contact with. This happens because in transmission spectroscopy, the absorbance *A*, scales as per the Lambert–Beer law [16], [17], proportional with the analyte concentration *c* and the optical path length *L*. Indirect spectroscopic approaches, such as photothermal spectroscopy, instead benefit from very small sample volumes. PTS is a sensitive technique used to indirectly measure optical absorption. In mid-IR PTS, a dual-beam configuration is commonly arranged: a sample is periodically illuminated by means of an excitation laser emitting in a spectral region where the target chemical/substance exhibits absorption feature. The mid-IR radiation gets absorbed and transduce in heating. A second laser is used to probe the thermal change in the sample under analysis, namely the probe laser. The reason why PTS is attractive is because, in general, the photoinduced temperature change Δ*T* scales proportional with the excitation laser power $P\left(\tilde{\nu }\right)$ and the optical absorption coefficient $\alpha \left(\tilde{\nu }\right)$ , and inversely to the square of the excitation beam radius *ω*_*e*_, the sample density *ρ* and heat capacity *C*_*P*_ (see eq. (1)) [18]. In this sense, using powerful laser sources (e.g., QCL and/or Distributed Feedback (DFB) lasers) [19], [20] and dealing with a miniaturized transducer, meaning reducing the area of interaction, enhance the resulting sensitivity.

(1) $$\Delta T\propto \frac{P\left(\tilde{\nu }\right)\alpha \left(\tilde{\nu }\right)}{\rho C_{P}\omega_{e}^{2}}$$

Several detection schemes have been reported in literature for targeting samples in different phases: gas, liquids, or solids [21], [22], [23], [24], [25], [26], [27]. Most of them use either free-space optical systems [21], [23], [24], [25], [27] or fiber optics [28], [29]. In fact, PTS would really benefit from miniaturization. By decreasing the test sample volume, not only the reagents, solvents, and waste volumes are reduced but the duration of the analysis is also shortened [18]. Vasiliev et al. have reported promising results about an on-chip PTS sensor using a Silicon-On-Insulator (SOI) suspended MRR for the detection of a photoresist patterned in the annular region [30]. In this work, we present a PTS on-chip sensor that foresees an easier fabrication process of the ring and other relevant differences. In detail, we use a Si_3_N_4_ MRR instead of a suspended Si ring, and for the analyte patterning, we simply spin-coated the polymer on top of the device, before cleaving. Additionally, instead of an Optical Parametric Oscillator (OPO), we exploit an EC-QCL for a wider spectral coverage of the excitation source and better qualitative spectra reconstruction as well as faster scan speed for PTS signal acquisition. We use a Cassegrain reflector to focus the pump beam instead of an optical fiber, avoiding optical losses and exploiting more optical power. Operating the excitation laser in pulsed mode, we do not need any temperature stabilization of laser head, additionally improving the overall system compactness. Pulsed excitation mode in PTS has been theoretically discussed in several works considering different materials and/or different dual-beam arrangement (e.g., collinear) [18], [31], [32], [33], [34]. The time profile of the pulsed beam $\Phi \left(\text{t}\right)$ can be expressed as:

(2) $$\Phi \left(\text{t}\right)=Qt_{0}e^{-{\left(t-\xi \right)}^{2}/\tau^{2}}$$

where *Q* is the pulse energy, *τ* represents the pulse width, *ξ* denotes the time to peak irradiance, and *t*_0_ is a normalization parameter [18]. An analytical solution for this specific case, where the sample is a multilayers WG, is unavailable in literature.

We report on a proof-of-concept photothermal sensor that relies on integrated optics. We combine a Si_3_N_4_ MRR transducer and a probe–pump schematic for PTS measurement of polymethylmethacrylate (PMMA). A 160 nm PMMA layer is spin-coated on top of a 33 µm radius MRR. In our setup, we use a tunable near-infrared (NIR) laser to tune the MRR at the inflection point (IP) of a picked resonance within the transmission spectrum. The IP of a resonance represents the point of the resonance profile, where the steepness reaches the highest value, leading to a maximized sensitivity in the presence of a resonant wavelength shift Δ*λ*_Res_. The employed EC-QCL emits between 1560 and 1770 cm^−1^, covering the spectral region in which PMMA absorbs (absorption peak at 1730 cm^−1^, 5.8 µm). PTS enables to gain a qualitative spectral information of the target analyte by sweeping the excitation laser on the chip and recording the lock-in amplifier (LIA) demodulated signal using the laser pulse rate as a reference trigger. The modulation frequency and pulse width of the excitation beam have been experimentally optimized to maximize the PTS signal amplitude of the MRR with the PMMA film. The PTS signal is recorded and normalized to the optical power of the excitation laser. The normalized PMMA PTS spectrum is compared with the FTIR spectrum. We also provide a concise overview of the transduction scheme, of the experimental setup, and determine the best operational parameters. Last, some comments, outlooks, and possible future works are presented as a conclusion.

## 2 Experimental section

### 2.1 Fabrication

**Figure 1:**
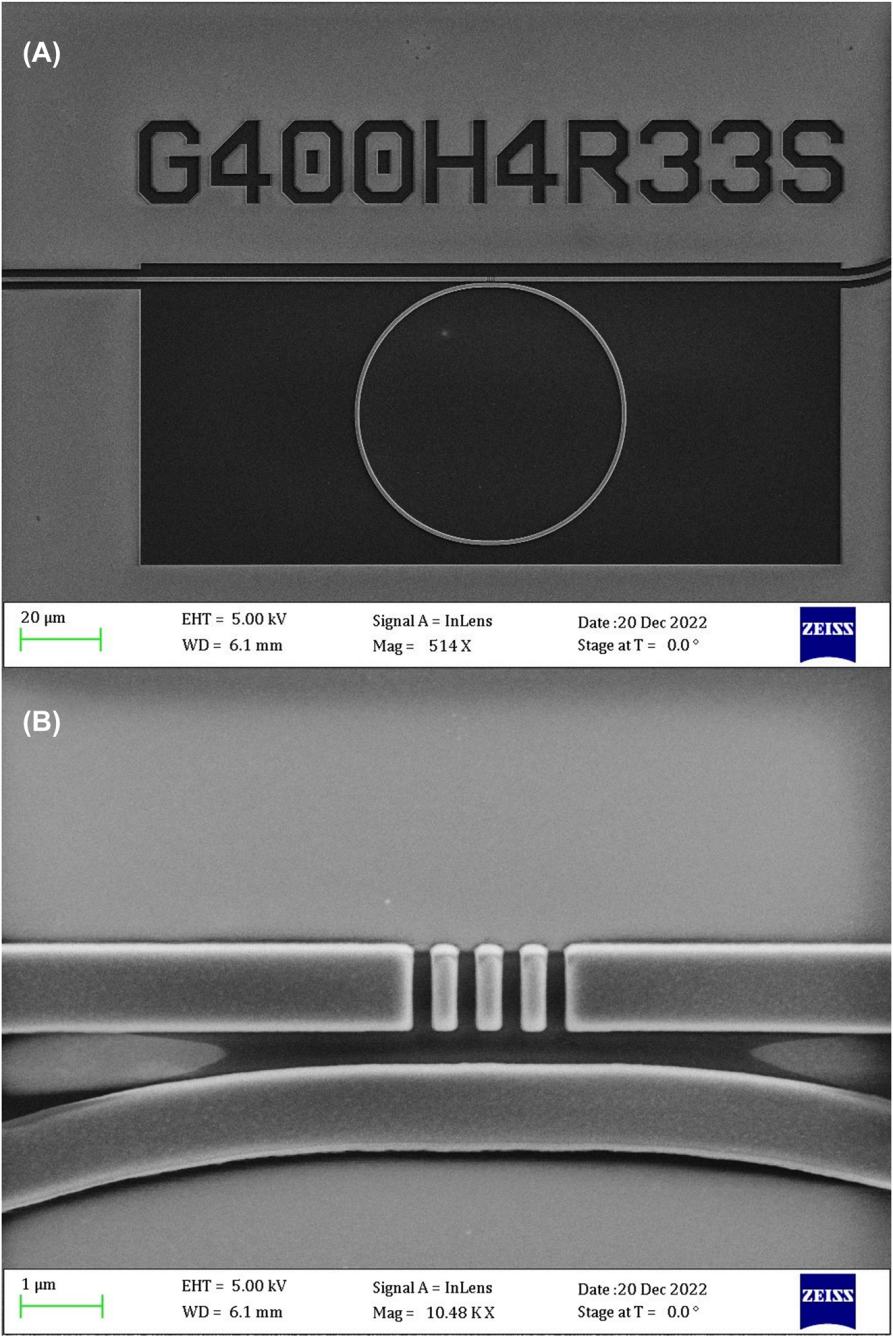
SEM images of the MRR employed for the PTS experiment. (A) The ID code indicates respectively: the gap of 400 nm in the coupling region between the straight WG and the ring itself, the number of PTE in the coupling region, in this case 4, the ring radius (33 µm); the S indicates the shape of the PTE, in this case, rectangular slot. (B) SEM image of a magnification of the coupling region with partially transmitting elements.

The air cladded MRRs were fabricated using a thermally oxidized 4″ bulk silicon wafer (BOx thickness of 2.2 µm) with a Si_3_N_4_ layer (300 nm thick), deposited with plasma-enhanced chemical vapor deposition (PECVD). First, a layer of ZEP 520A resist (∼450 nm thick) was spin-coated on the wafer. The device layouts were patterned on the resist by means of 100 kV electron beam lithography (EBL) and developed in a bath of n-amyl acetate solution for 90s and rinsed with IPA. The patterns were transferred to the PECVD Si_3_N_4_ layer through inductively coupled plasma (ICP) etch step with CHF_3_:O_2_ chemistry in a 21:4 ratio (etch rate of ∼1.5 nm/s). The residual resist was later removed through an O_2_ plasma ashing step and a bath in 1165 Remover for 10 min. For the analyte deposition, a layer of PMMA-A4 (160 nm) was spin-coated on top of patterned Si_3_N_4_ layer. The thickness of the PMMA film was achieved following the spin curve of the polymer and experimentally measured with a profilometer. Among the different MRRs design and geometry, better described in one of our work [35], [36], the one reported in the Scanning Electron Microscope (SEM) image (Figure 1A and B) have been employed to perform the here-presented PTS experiment. The ring presents four partially transmitting elements (PTE) aiming for enabling the realization of a Fano-line shape, steeper, and sharper to maximize the sensitivity of the PTS signal [37]

### 2.2 Experimental setup and measurement procedure

For optical characterization of WGs, an End-Fire (EF) setup [38] was home-built. It allows for butt-coupling the light to one facet of the WG and retrieves the light in output from the other facet. The setup includes a polarizing beam splitter that transmits only TE-polarized light to avoid optical higher order modes propagation. The sample holder is an aluminum block that includes a Peltier element for temperature stabilization (20 °C). An Optical Spectrum Analyzer (Yenista Optics, OSA20) 20″ was used to acquire the device’s transmission spectrum in a range between 1560 and 1610 nm. The highest sensitivity allowed from the instrument was employed for spectrum trace acquisition (0.5 nm/s). A NIR photodiode sensor (Thorlabs, S155C) was used to measure the output power. The alignment was performed using an InGaAs camera (Electrophysics, MicronViewer 7290A) for monitoring of light propagation along the WG.

One resonance was picked from the transmission spectrum of the WG, and the inflection point was roughly found from OSA trace. The probe laser is a tunable laser (Yenista Optics TLS, tunable laser between 1500 and 1630 nm) tuned at the inflection point. The wavelength was finely adjusted while performing PTS experiment using a resolution of pm range in fine-tuning mode.

**Figure 2:**
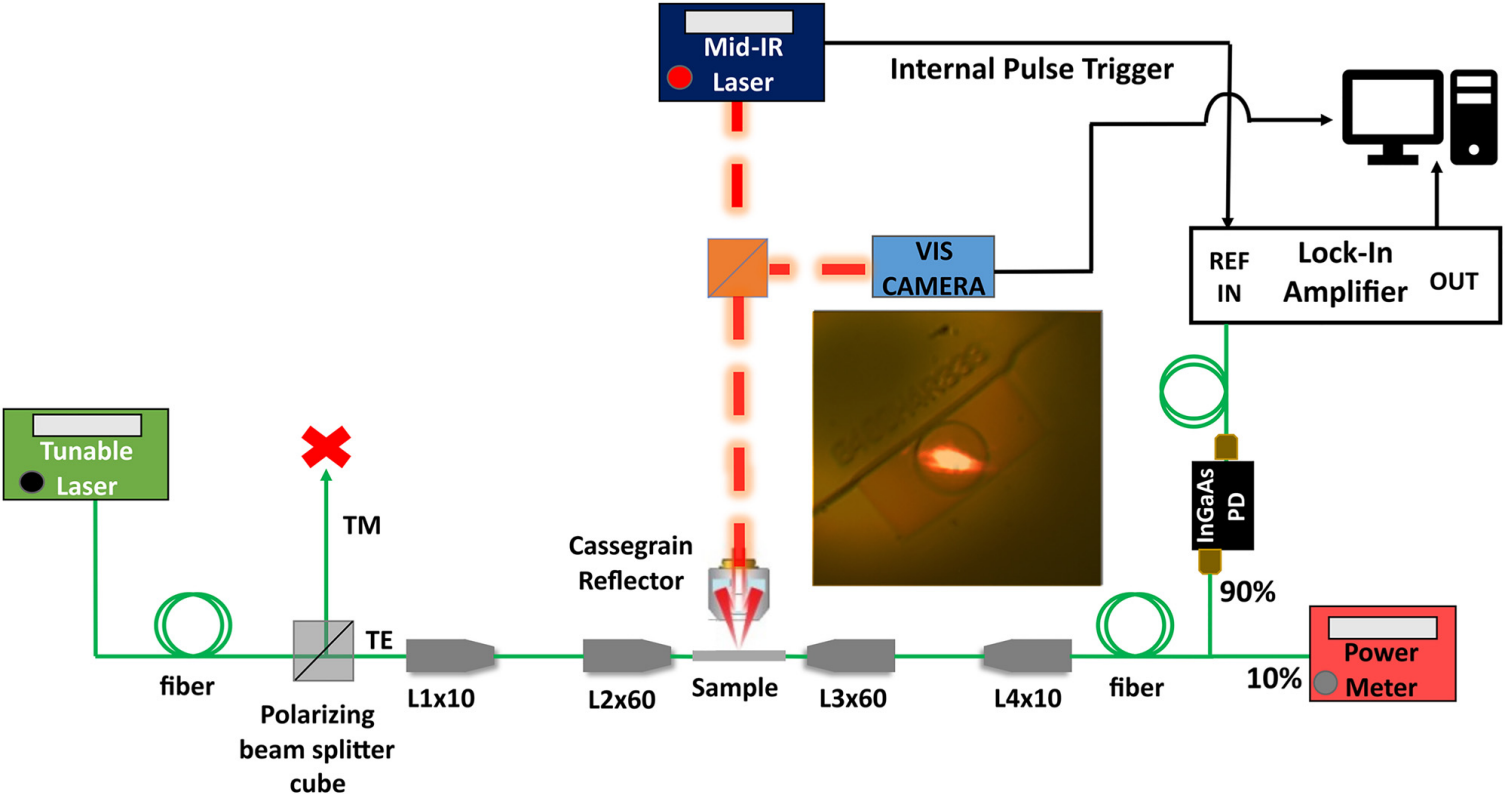
EF variation to perform PTS experiment. The NIR camera is substituted by visible imaging system, used to align the mid-IR excitation laser on top of the ring via visible laser embedded in EC-QCL head.

To perform the PTS measurement, depicted in Figure 2, the NIR imaging system, prior used for butt-coupling, was replaced by a visible camera (Thorlabs, DCC1645C) in combination with a beam-combiner (Thorlabs, CM1-BP145B4 45:55 (R:T), 3–5 µm). In this way, it was possible to align the mid-IR laser (DRS Daylight Solutions, MIRCAT) on top of the MRR to which the probe was coupled. This is made possible by means of a visible laser embedded in the mid-IR source head. The red laser was positioned at the center of the ring resonator. Due to a possible mismatch between the alignment laser and the proper IR beam, finer adjustment of the beam position on top of the ring was made along the *x*–*y*–*z* axis while performing PTS experiment.

The collimated mid-IR beam was focused on the MRR by means of a Cassegrain reflector (Thorlabs, LMM25X-P01) with a magnification of 25×, a numerical aperture N.A. of 0.4, and a focal length *f* of 8 mm. The reflective microscope objective ensured the excitation laser beam radius *ω*_*e*_ to be shrunk (measured *w*_0*e*_ at the focal point ∼16 µm) such that the mid-IR radiation could be concentrated on top of the MRR. This in turn maximizes the temperature rise in the PMMA deposited on the responsive region: probe–pump beam volume of interaction.

The probe output signal from the WG was sent to a fiber-coupled InGaAs photodetector (Thorlabs, DET08CFC/M). The signal from the detector was sent as an input to a LIA (Anfatec, USB LockIn 250), which demodulates it, with a time constant of 100 ms, taking the internal pulse trigger from the mid-IR laser as a reference. The raw demodulated output from LIA constitutes the actual measured PTS signal.

The excitation source was operated in pulsed mode, with a forward sweep scan resolution of 5 cm^−1^/step. Pulse width and rate were experimentally optimized, as described in “Results and discussion” section.

The magnitude of the signal vector of the LIA demodulated PTS signal was normalized to the mid-IR source optical power spectrum profile recorded with a mercury cadmium telluride (MCT) detector (DRS Daylight Solutions, Amplified MCT detector) at the sample position. This allows to reconstruct the PMMA absorption spectrum for evaluation of peak shape and positioning. A Savitzky–Golay filter was used to remove a prominent water vapor contribution. See the recorded spectra reported in the “Results and discussion” section.

## 3 Results and discussion

### 3.1 The transduction scheme

In this work, a Si_3_N_4_ MRR is employed as a transducer. It is important to underline that the design/geometry optimization and the theoretical physical background of ring resonators are out of the scope of this study. More details can be found in one of our works [36]. The main goal of our work is to demonstrate that Photonic Integrated Circuits (PICs) can be exploited and further explored for PTS sensing in a compact format, sensitive sample detection, and fast response time, using a MRR as an example. Similar conclusions and comments also apply to other waveguide-based transducers, such as Mach–Zehnder Interferometers (MZIs), Photonic Crystal Cavities (PhCs), surface-plasmon resonance (SPR) waveguides [39], [40], etc., depending on the corresponding working principle.

**Figure 3:**
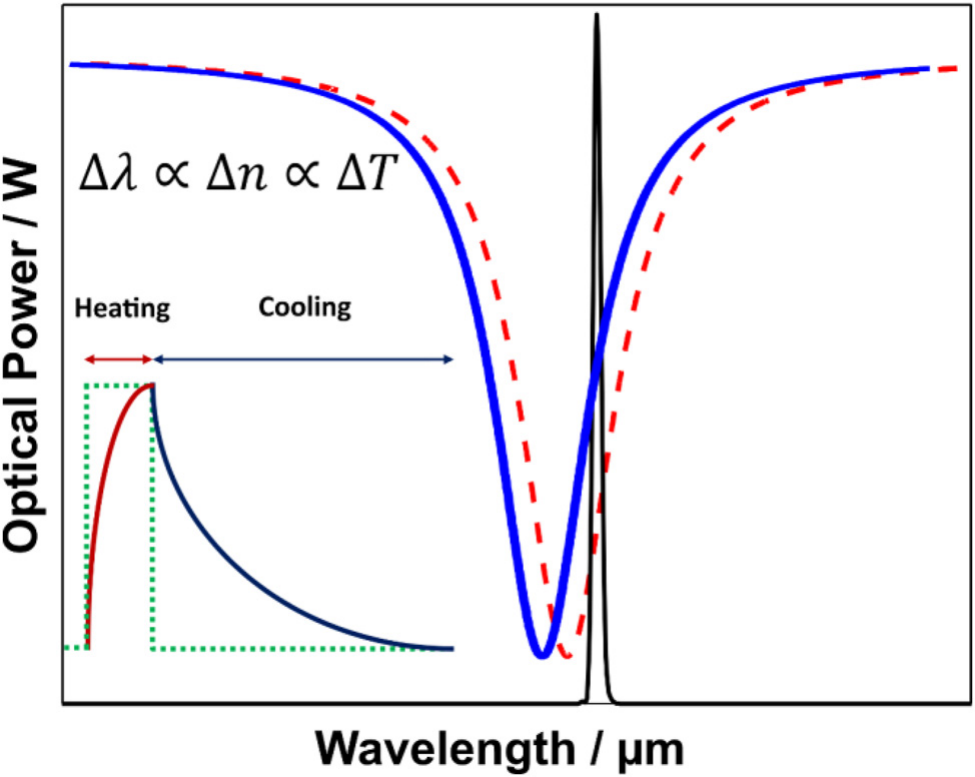
Transduction scheme. The probe laser is tuned at the inflection point, aiming for highest sensitivity, linearity, and PTS signal maximization. The excitation source is enabled to sweep, inducing a *λ*_Res_, due to temperature change Δ*T* (proportional to the absorption of the analyte *α*) and to a consequent overall effective refractive index change Δ*n*_eff_.

In this scenario, using a MRR as a transducer, the detection variable is the resonant wavelength shift Δ*λ*_Res_. By tuning the probe laser at the IP, we aim for retrieving highly sensitive shift of the resonant wavelength photoinduced by the periodical heating and cooling of the MRR, as a function of excitation laser power $P\left(\tilde{\nu }\right)$ . The transduction scheme is depicted in Figure 3.

After the MRR is covered with PMMA, the refractive index of the cladding *n*_clad_ changes from *n*_air_ = 1 to *n*_PMMA_ = 1.48 and a Δ*λ*_Res_ occurs.

Once we perform a PTS experiment, the PMMA-cladded WG resonances’ position become a baseline and a new shift will occur as soon as the excitation source is enabled, and a temperature change happens consequently.

### 3.2 The photothermal spectrum recorded with MRR

**Figure 4:**
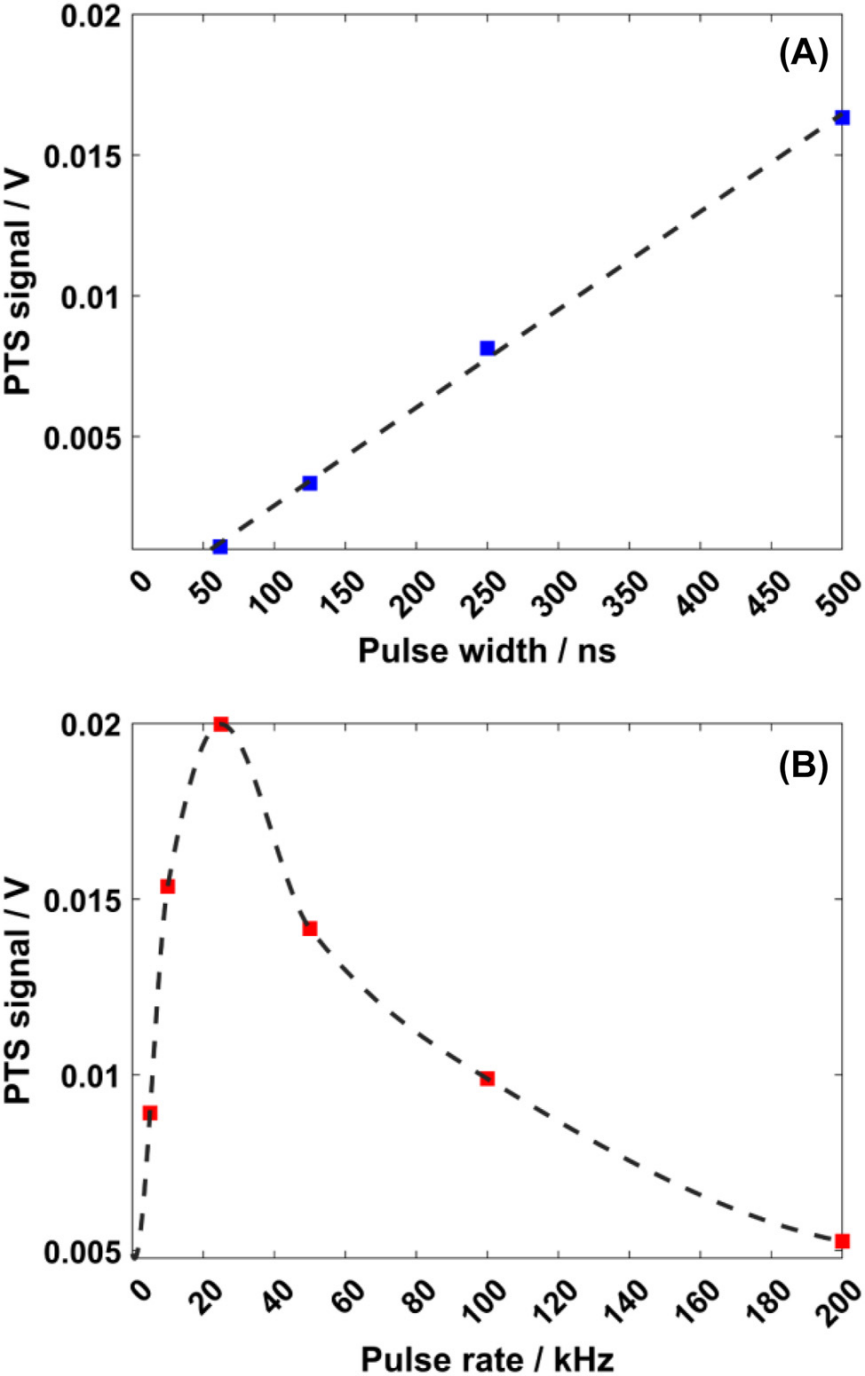
PTS excitation laser tuning for optimal pulse width and pulse rate. (A) PTS signal at PMMA absorption peak (∼1730 cm^−1^) recorded by fixing the repetition rate at 50 kHz and tuning the pulse width. (B) PTS signal at PMMA absorption peak (∼1730 cm^−1^) recorded by fixing the pulse width at 500 ns and tuning the pulse rate.

Using EF setup, the transmission spectrum of the MRR was recorded. One resonance was picked for performing the PTS experiment (*λ*_Res_ = 1593.4 nm) and the IP was roughly identified from the OSA acquisition. The probe laser was coupled to the WG and tuned to the selected IP. By removing the NIR camera and sliding in the Cassegrain reflector system, the PTS measurement could be performed. Before sweeping the EC-QCL, the mid-IR laser was tuned at the absorption peak of PMMA (∼1730 cm^−1^) for signal optimization. It consisted in (I) finding the optimal excitation pulse rate and pulse width, (II) fine adjustment of the probe laser wavelength at the IP, and (III) fine adjustment of the mid-IR beam position on top of the ring by changing the *x*–*y*–*z* position of the Cassegrain after switching from visible alignment laser to IR beam. An optimal duty cycle of 1.25 % was found for the first optimization, meaning a pulse rate of 25 kHz and a pulse width of 500 ns, the highest allowed from excitation source settings (see Figure 4A and B). The optimal duty cycle corresponds to the full device thermal relaxation time to reach the equilibrium temperature. The different layers get heated up during the mid-IR laser pulse and a longer duration time is needed to bring the MRR to the initial temperature before enabling for the next excitation pulse.

**Figure 5:**
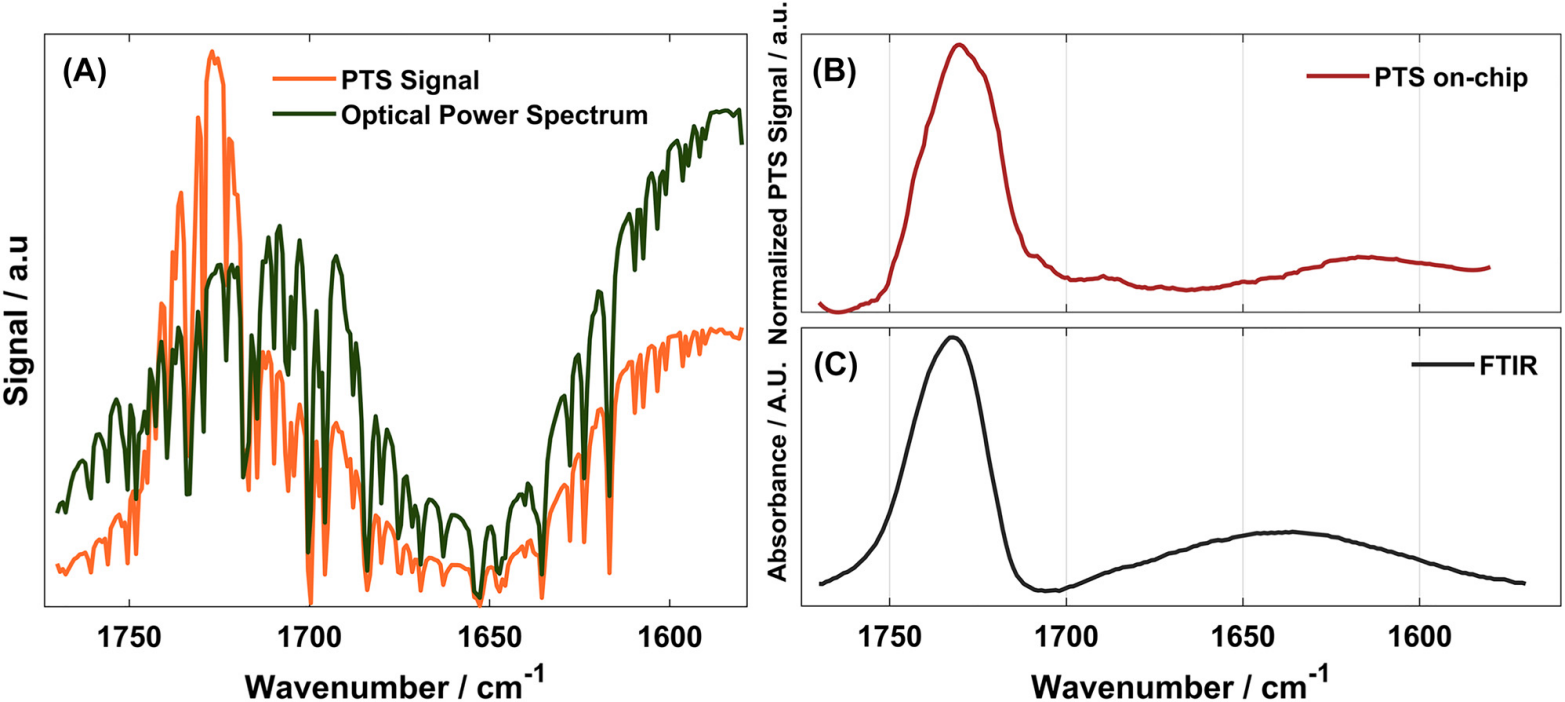
Mid-IR spectra of PMMA recorded with PTS on-chip setup. (A) In orange, the raw LIA PTS signal of the PMMA-coated Si_3_N_4_ MRR (averaged of 10 scans). In dark green, the optical power spectrum of the EC-QCL between 1580 and 1770 cm^−1^ recorded with an MCT detector at the sample position. (B) Normalized PTS signal obtained by division of the orange and the green acquisition. (C) FTIR reference spectrum of PMMA-coated Si_3_N_4_ MRR.

Using the optimal parameters and alignment, we recorded 10 PTS spectra using forward sweep scan mode with a resolution of 5 cm^−1^/step and subsequently averaged (acquisition scan ∼ few seconds each). Using an MCT detector, we recorded the optical power spectrum of the excitation laser at the sample position, immediately before sample interaction. The PMMA absorption feature was reconstructed, by normalizing PTS signal to MCT acquisition, and compared against FTIR spectrometer (PerkinElmer Spectrum 400) absorbance spectrum. In Figure 5A, the spectra are reported. Water vapor lines are pronounced in both acquisitions, and they are overlapped across the whole spectral range. When performing normalization between the PTS and the power spectrum, a Savitzky–Golay filtering was additionally performed to remove water artefact. The qualitative absorption spectrum of PMMA, obtained via photothermal spectroscopy on-chip sensor, is depicted in Figure 5B. The PMMA absorption peak is at about 1730 cm^−1^ and it is in good agreement with the reference one retrieved with commercial FTIR (see Figure 5C). The reference measurement was performed by using a Si_3_N_4_ air-cladded wafer piece as a background and a Si_3_N_4_ PMMA-coated wafer.

### 3.3 The photothermal effect in the MRR

**Figure 6:**
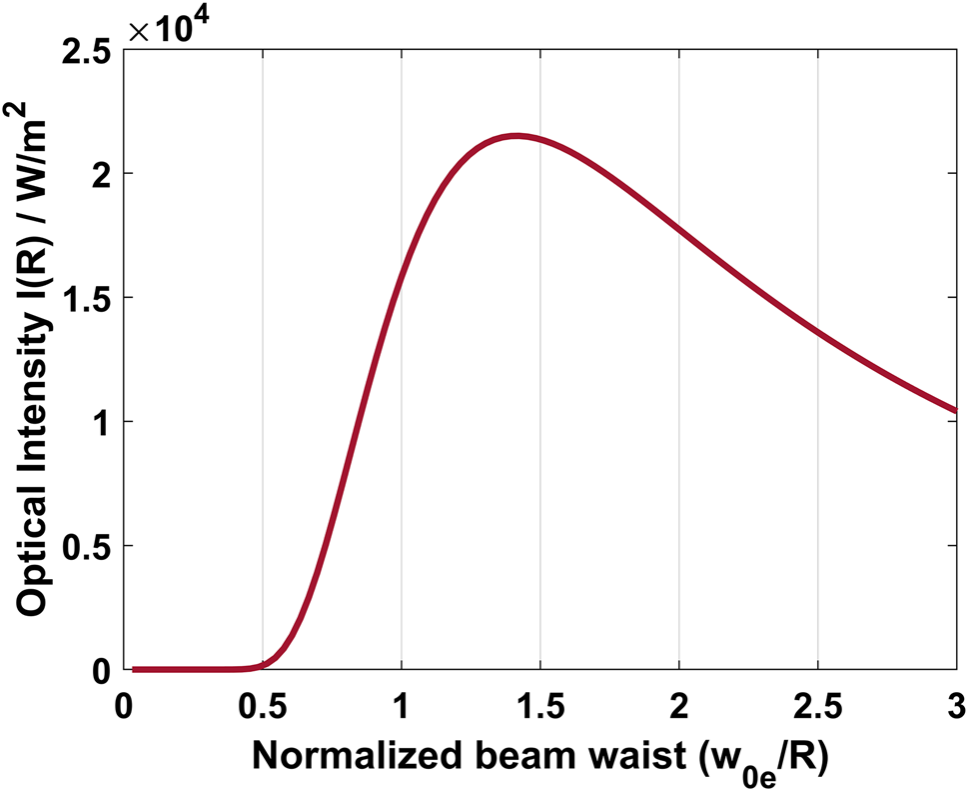
Optical intensity [W/m^2^] at the ring position (*r* = *R* = 33 μm) as a function of the excitation beam waist radius.

**Figure 7:**
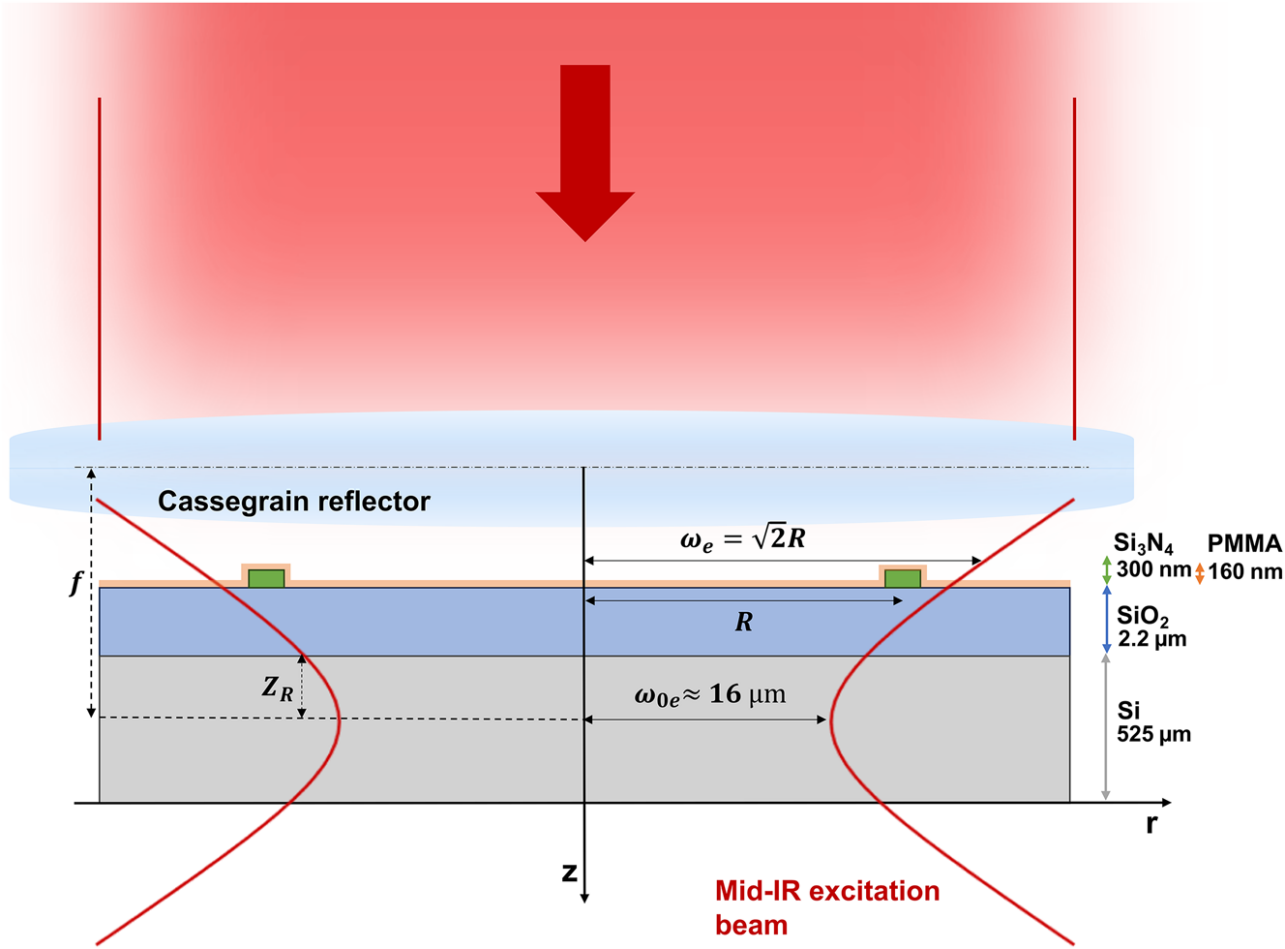
The pulsed mid-IR excitation beam arrives collimated at the Cassegrain reflector. The Gaussian beam penetrates the sample. The optimal beam radius condition is achieved when the Cassegrain position is moved along the *z*-axis from *ω*_0*e*_ (at the focal length *f*) to $\omega_{e}=\sqrt{2}R$ (off-focus).

The periodical heating and cooling generated by the excitation laser in the sample can be translated into a change of the overall effective refractive index Δ*n*_eff_ of the WG defined as follows [41]:

(3) $$\begin{array}{ll} \Delta n_{\text{eff}} & =\Gamma_{SiO_{2}}{\left(\frac{\delta n}{\delta T}\right)}_{SiO_{2}}\delta T_{SiO_{2}}+\Gamma_{Si_{3}N_{4}}{\left(\frac{\delta n}{\delta T}\right)}_{Si_{3}N_{4}}\\ & \;\times \delta T_{Si_{3}N_{4}}+\Gamma_{\text{PMMA}}{\left(\frac{\delta n}{\delta T}\right)}_{\text{PMMA}}\delta T_{\text{PMMA}} \end{array}$$

where Γ_*x*_ represent the confinement factors of the guided mode in the substrate (SiO_2_), in the core (Si_3_N_4_), and in the cladding (PMMA), respectively, ${\left(\frac{\delta n}{\delta T}\right)}_{x}$ are the thermo-optical coefficients (TOCs) of the different materials, and *δT*_*x*_ are the temperature changes in each of the sections, where also the absorption $\alpha \left(\tilde{\nu }\right)$ contribution is taken into account. However, in such a complex configuration, it is difficult to predict the overall temperature gradient since each layer has different thickness, thermal properties, absorption coefficient, and in turn an optical power decay of excitation due to absorption from a layer to the following must be included as well. The excitation source, with a Gaussian radial profile, is focused on the center of the ring, as optimally aligned in the experimental measurement. The sensitive area is at the ring annular perimeter, where probe and pump beam lasers overlap. The optical intensity $I\left(r\right)$ of the beam with an optical power *P* can be described with a Gaussian function $I\left(r\right)=\left(2P/\pi \omega_{e}^{2}\right)e^{\left(-2r^{2}/\omega_{e}^{2}\right)}$ . Figure 6 shows the optical intensity at the ring position (*r* = *R* = 33 μm) as a function of the excitation beam radius (*ω*_*e*_). Assuming that the beam is centered, it is observed that the maximum optical intensity at the ring position occurs for $\omega_{e}=\sqrt{2}R∼46.7\;\mu \text{m}$ . Therefore, in this condition, a maximum temperature variation is obtained within the ring and surroundings, maximizing the amplitude of the PTS signal. After the excitation pulse, the heat absorbed mainly by the PMMA and partially by the core (Si_3_N_4_) and the substrate (SiO_2_) diffuses throughout the sensor, returning to the thermal equilibrium condition. During the measurement, as described in the section “The photothermal spectrum recorded with MRR,” optimization of the PTS (III) consisted in adjustment of the Cassegrain system position on top of the ring along the three axes. By moving the *z* position, the beam width changed consequently being off-focus, and a maximized PTS signal was achieved. Figure 7 depicts the interaction region between the mid-IR excitation laser beam and the MRR when operating at the maximum optical intensity at the ring position. For an excitation beam with a radius much smaller than the ring, the center of the ring will have a high temperature, but the WG region will not be heated considerably. If the excitation radius is much larger than the ring radius, the light intensity is spread over a large area, heating a region much larger than the ring. The optimized condition occurs for the excitation beam radius equal to $\sqrt{2}$ of the ring radius (see Figures 6 and 7).

A resonance shift *dλ*_Res_ can be approximately estimated following eq. (4) [42]:

(4) $$d\lambda_{\text{Res}}=\lambda_{\text{Res}}\left(\frac{\Delta n_{\text{eff}}}{n_{\text{eff}}}+\Sigma \delta T_{x}\alpha_{\mathrm{THx}}\right)$$

The approximated shift *dλ*_Res_ due to the PTS thermal change in the sample is in the order of few pm. It mainly depends on the refractive index change Δ*n* that occurs because of the thermal change *δT*_*x*_. It scales with the TOCs slope of each layer. The overall change in the WG translates in an effective refractive index change Δ*n*_eff_ (see eq. (3)). An increase in temperature also leads to an expansion proportional to the thermal expansion coefficient of each material *α*_*THx*_ due to the respective *δT*_*x*_. Eq. (4) takes into account both the optical change of the guided mode and the physical thermal expansion photoinduced by PTS in the WG. The thermal change can be seen as a weighted contribution of involved TOCs of the WG layers. In fact, the radiation, arriving from the top, reaches the PMMA layer that has a certain thickness *L*_1_ (160 nm). The intensity gets absorbed and decay exponentially due to absorption within *L*_1_ ( $I_{1}=I_{0}e^{-\alpha_{\text{PMMA}}L_{1}}$ _)_. Since PMMA has a negative TOC, the contribution due to increasing temperature in the cladding shifts the resonance toward blue. The remaining optical intensity that reaches the core, immediately below *L*_1_, slightly heats up the 300 nm (*L*_2_) Si_3_N_4_ as well ( $I_{2}=I_{1}e^{-\alpha_{Si_{3}N_{4}}L_{2}}$ ), leading to a resonance shift to the opposite direction (red) due to the positive TOC of Si_3_N_4_. The same effect applies to the substrate based on the residual intensity. It is important to address that the WG materials, Si_3_N_4_, as well as the substrate (SiO_2_ and Si), have an optical absorption coefficient *α* much lower than the absorption peak of PMMA (at 1730 cm^−1^) and can be neglected (see Table 1). The absorption coefficients *α* have been taken from database available in literature [43]. The PTS method presented in this work allows qualitative measurement of the absorption spectrum of the thin film in contact with the MRR. However, it cannot provide a direct absolute absorption coefficient measure. The PTS signal is proportional to the absorbance, meaning that the optical absorption could be obtained only after validation against reference methods and sensor calibration. A critical PMMA thickness *L*_1_ could even lead to an athermal condition where the blue shift from the analyte is fully compensated by the red shift from the core. In this scenario, no detectable shift could be measured. Temperature insensitive devices’ design is widely exploited when PICs thermal shifts must be avoided and either negative TOCs for the substrate are used or certain polymers with negative TOCs are coated on top of the WG by thickness optimization [42], [44], [45], [46].

**Table 1:** Relevant parameters of MRR and PTS setup.

Parameter	Value	Unit
Radius	33	µm
Q-factor	∼12 K	–
$Resonance\;slope\;\frac{dP}{d\lambda }$	1.13 × 10^−4^	µW/nm
*P*_probe,IP_	1.2	µW
$P_{pump,1730\text{c}{\text{m}}^{-1}}$	200	µW
*λ*_Res_	1593.44	nm
$\alpha_{\text{PMMA}}\left(1730\;\text{c}{\text{m}}^{-1}\right)$ [43]	7816	cm^−1^
$\alpha_{Si_{3}N_{4}}\left(1730\;\text{c}{\text{m}}^{-1}\right)$ [43]	448	cm^−1^
$\alpha_{SiO_{2}}\left(1730\;\text{c}{\text{m}}^{-1}\right)$ [43]	29.6	cm^−1^
$\alpha_{Si}\left(1730\;\text{c}{\text{m}}^{-1}\right)$ [43]	0.03	cm^−1^
*L*_PMMA_	160	nm
LIA integration time *τ*	100	ms
PMMA *δn*/*δT* [45]	−1.3 × 10^−4^	K^−1^
Si_3_N_4_ *δn*/*δT* [44], [45]	2.45 × 10^−5^	K^−1^
SiO_2_ *δn*/*δT* [44], [45]	1 × 10^−5^	K^−1^

### 3.4 Normalized Noise Equivalent Absorption coefficient

Apart from the excitation source parameters’ optimization, what matters the most in PTS sensitivity enhancement is the shape of resonance used in the transduction scheme, the greater the steepness, and the more sensitive the measurement. Moreover, the choice of materials and their thermal sensitivity, meaning their TOCs, must be cleverly engineered to promote target analyte excitation amplification.

Considering a polymer, with concentration *c* = 100 %, a noise equivalent thickness (NET) can be estimated as a figure of merit. It could be expressed as the ratio between the PMMA thickness *L*_1_ (160 nm) and the calculated signal-to-noise-ratio (SNR) of the system, considering the 1*σ* retrieved from the averaged replicates of the PTS spectra acquisition. A minimum detectable absorption coefficient *α*_min_ is evaluated as:

(5) $$\alpha_{\min}\left(\tilde{\nu }\right)=\frac{NET\left(\tilde{\nu }\right)\cdot \alpha_{\text{PMMA}}\left(\tilde{\nu }\right)}{1\;\text{cm}}$$

A Normalized Noise Equivalent Absorption coefficient (NNEA) can be estimated as:

(6) $$NNEA\left(\tilde{\nu }\right)=\frac{P_{\text{excitation}}\left(\tilde{\nu }\right)\cdot \alpha_{\min}\left(\tilde{\nu }\right)}{\sqrt{\Delta }f}$$

The obtained NNEA (1730 cm^−1^) is found to be 6.11 × 10^−6^ cm^−1^ W/Hz^1/2^. The achieved value is comparable to the one obtained in Vasiliev et al. work [30]. It is worth noting that in our case, dealing with Si_3_N_4_ as a core, rather than Si [30], less power limitations due to thermal nonlinearities are encountered meaning that NNEA value could be further improved even increasing the probe laser power aiming for higher SNR. For a summary of the most relevant parameters of the MRR and of the PTS experiment, please refer to Table 1.

## 4 Conclusions and outlook

In this work, we report on a photothermal waveguide-based sensor capable of measuring the optical absorption spectrum of a thin film in contact with the chip, allowing the determination of the polymer absorption spectrum. Aiming for compactness and fast analysis time, we have demonstrated that PTS allows for sensor miniaturization by reducing the sample volume and enhancing the generated thermal change in the sample. Operating the powerful EC-QCL in pulsed mode, we avoid the need of temperature cooling of the laser head, usually operated in CW mode for low frequencies of modulation adopted in PTS. This in turn leads to increased sensor mobility. Our analysis takes few seconds for a full scan, and we cover a broad spectral region using the PMMA absorption feature as an example. By cleverly playing and tuning the design and the materials’ properties, avoiding a thermalization condition, a highly sensitive transducer can be developed, and higher limits of detection can be achieved. The obtained qualitative spectrum of PMMA is in excellent agreement with the one measured with FTIR spectrometer. The technique is versatile and can be applied to different phase target analytes only by replacing the excitation source. Not only the pump laser can be substituted but also a different transducer principle could be employed such as MZIs where longer arms of the interferometer could be designed to increase the probe–pump interaction. For instance, in the scenario of liquid phase analytes, a bottom excitation configuration could be adopted such that the analyte optical path length limitations due to strong matrix absorption can be overcome and a further compact setup can be built. Rooms for improvement could be better control of environmental conditions, probe-laser coupling instability reduction via some NIR permanent coupling solutions of integration (fiber arrays or grating coupler plus quasi-planar fiber arrays), locking-scheme of the probe laser at the inflection point avoiding power fluctuations, microfluidics (lab-on chip) for the specific case of liquid samples in order to inject the proper amount of sample, and flush the surface between one concentration and the next.
